# Selected Endemic Zoonoses in Pigs Presenting for Slaughter in Kampala, Uganda

**DOI:** 10.4269/ajtmh.20-0033

**Published:** 2020-10-12

**Authors:** Christine Atherstone, Georgies F. Mgode, Navneet K. Dhand, Silvia Alonso, Delia Grace, Michael P. Ward, Siobhan M. Mor

**Affiliations:** 1 Sydney School of Veterinary Science, The University of Sydney, Camperdown, Australia;; 2 Pest Management Centre, Sokoine University of Agriculture, Morogoro, Tanzania;; 3 International Livestock Research Institute, Addis Ababa, Ethiopia;; 4 International Livestock Research Institute, Nairobi, Kenya;; 5 Natural Resources Institute, University of Greenwich, Kent, United Kingdom;; 6 Institute of Infection and Global Health, University of Liverpool, Liverpool, United Kingdom

## Abstract

Leptospirosis, brucellosis, and Q fever (coxiellosis) are bacterial zoonoses that cause acute febrile illness in people as well as reproductive losses in pigs. Pig keeping is an increasingly important livelihood to millions of smallholder farmers in Uganda because of exponential increases in demand for pork. The prevalence of leptospirosis and Q fever in pigs is unknown, and the few studies of porcine brucellosis have estimated a range of seroprevalence. Therefore, we undertook a prevalence survey of leptospirosis, brucellosis, and Q fever in pigs using quantitative real-time PCR to determine the potential importance of these zoonoses to the growing pig sector in Uganda. Six hundred forty-nine pigs were sampled in 2015–2016 at an urban pork slaughterhouse. Ten percent of pigs (*n* = 68) had leptospiral DNA in either their kidney or reproductive tissue. In adjusted analyses, variables predictive of leptospiral status included female sex (odds ratio [OR]: 2.37, *P* < 0.01) and pigs sampled in March 2016 (OR: 2.23, *P* = 0.02) and October 2016 (OR: 0.30, *P* = 0.04). DNA fingerprinting revealed circulation of at least four distinct serovars in these pigs. *Brucella* spp. and *Coxiella burnetii* DNA were not detected in any sampled pig. This is the first report of widespread circulation of pathogenic *Leptospira* spp. in pigs in Uganda, suggesting that leptospirosis likely has a greater impact on the health of pigs than was previously recognized. Pig farmers, pig traders, and slaughterhouse workers may be at greatest occupational risk because of their direct contact with infective leptospires in aborted fetuses, bodily fluids, and other tissues.

## INTRODUCTION

As control efforts have led to significant decreases in malaria throughout sub-Saharan Africa,^1^ the diagnosis, treatment, and control of non-malarial febrile illnesses are gaining overdue attention as public health priorities.^2–4^ In Tanzania, for instance, acute bacterial zoonoses were a frequent cause of presentation for severe febrile illness (26.2%), with leptospirosis, brucellosis, and Q fever, among the most common bacterial causes.^5^ These three zoonoses pose a dual burden, affecting the health and well-being of millions of livestock keepers in sub-Saharan Africa, while also causing significant economic losses because of the impact of infection in their livestock.^2,4,6^ Although human cases of leptospirosis,^5,7–9^ brucellosis,^5,10,11^ and Q fever^5,12,13^ in East Africa confirm that these bacterial infections are present, the diagnosis and control of infection in livestock remains scarce.

To date, most of the research on zoonotic bacterial infections in livestock in sub-Saharan Africa has focused on ruminants.^2,4,6^ However, pig keeping has become an increasingly important livelihood strategy in parts of sub-Saharan Africa, particularly in Uganda.^14^ The incentive for farmers to raise pigs is driven by the growing consumption of pork,^15^ projected to increase by 237% between 2000 and 2030.^16^ This growth in pork consumption is not unique to Uganda; increased consumption is also projected in Kenya, Tanzania, and the Democratic Republic of Congo.^16^ Despite this massive growth in pig keeping and pork consumption, data are scarce on the incidence and burden of zoonotic diseases in pigs, making it difficult to develop a systematic strategy for disease control and intervention efforts.

Recent estimates rank leptospirosis as the leading zoonotic cause of mortality and morbidity in humans globally, with 1.03 million cases and 58,900 deaths each year.^17^ Without treatment, leptospirosis (caused by pathogenic *Leptospira* spp.) can cause renal failure, meningitis, and death.^18^ Tropical environments favor the transmission of *Leptospira* spp., where high seasonal rainfall, high temperatures, and high humidity allow the organism to survive for long periods of time in the environment.^19^ Other risk factors for infection include urbanization and population growth which place people in closer contact with each other and with animals, particularly rodent reservoirs.^17^ In Uganda, there is some evidence that leptospires are circulating in cattle and wildlife^7,20,21^; however, the burden of leptospirosis in other species, such as pigs, is unknown.

Brucellosis (caused by *Brucella* spp.) causes a severe debilitating illness in humans, with fever, sweats, fatigue, weight loss, headache, and joint pain persisting for weeks to months.^22^ Globally, there are a half million new cases of human brucellosis each year.^23^ *Brucella abortus*, *Brucella melitensis*, and *Brucella suis* cause abortion and infertility in their natural livestock hosts.^24^ Brucellosis is primarily acquired through direct contact with infected animals, by eating or drinking contaminated animal products, or by inhaling aerosolized material. The epidemiology of brucellosis in livestock in Africa is best understood for bovine brucellosis and to a lesser degree for caprine and ovine brucellosis.^6^ Although porcine brucellosis is suspected to be widespread,^25,26^ the epidemiology in pigs is poorly understood.^6^

Acute Q fever (caused by *Coxiella burnetii*) can lead to pneumonia, hepatitis, and death in humans, as well as miscarriage in pregnant women.^27^ Exposure to the bacterium is primarily from direct contact with infected reproductive tissues and other animal products^28^ or inhalation of aerosols from contaminated soil or animal waste.^29^ In animals, infection with *C. burnetii* (called coxiellosis) can lead to abortion and reduced reproductive efficiency; however, asymptomatic infections also occur in sheep, goats, and cattle.^30,31^ A recent review of Q fever epidemiology in Africa found evidence of infection in ruminants in many regions but no studies on the burden of infection or risk factors for animal disease in the East African region.^2^

Detection of leptospirosis, brucellosis, and coxiellosis in pigs is challenging for several reasons. The microscopic agglutination test (MAT) is the gold standard test for detecting antibodies to leptospires.^32^ However, in the absence of locally circulating serovars for inclusion in the MAT antigen panel, this test can vastly underestimate the prevalence of leptospirosis.^20^ There are several serological tests used to screen cattle and small ruminants for brucellosis, although the sensitivity and specificity of these serological tests in pigs have been found to be much lower than those in ruminants.^33^ Furthermore, to our knowledge, no serological assay has been validated for *C. burnetii* detection in pigs. Quantitative real-time PCR (qPCR) allows for rapid, specific identification of these pathogens, while also limiting the risk of laboratory-acquired infections associated with culture-based methods. Therefore, we undertook a prevalence survey of leptospirosis, brucellosis, and coxiellosis in pigs using qPCR to determine the potential importance of these zoonoses to the growing pig sector in Uganda.

## MATERIALS AND METHODS

### Ethics approvals.

Animal ethics approval for this research was obtained from the International Livestock Research Institute, Nairobi, Kenya (ILRI-IREC2015-01). The International Livestock Research Institute complies with United Kingdom’s Animals (Scientific Procedures) Act 1986 which contains guidelines and codes of practice for the care and use of animals used in scientific research. In addition, ethics approval was received from the Ugandan National Council for Science and Technology (A499) and Makerere University College of Veterinary Medicine, Animal Resources and Biosecurity (COVAB), Kampala, Uganda (SBLS.CA.2016). The Animal Ethics Committee at the University of Sydney, Australia, was also notified of external ethics approval (2015/891).

### Study area and rationale.

Wambizzi Cooperative Society, located in Nalukolongo, southwestern Kampala, is the only registered pork slaughterhouse in Uganda. It receives many pigs from a large area of the country to meet the urban demand for pork in Kampala and its surroundings.^34^ The large volume of pigs and the wide geographic area that these pigs are sourced from make it an ideal location for zoonosis surveillance.

### Study design and sample size.

We undertook a series of cross-sectional surveys during four discrete sampling periods between December 2015 and October 2016. The four sampling periods coincided with times when the number of pigs being processed was known to increase to meet pork demand during holiday times.^35^ Findings reported here were part of a larger study designed to demonstrate proof of freedom from filoviruses. Thus, samples from 649 pigs were available for detection of bacterial pathogens in the present study. This exceeds the minimum sample size (*n* = 381) needed to assess the apparent prevalence of these pathogens (based on an assumed prevalence of 1%,^36^ 95% confidence, and desired precision of ±1%).

### Selection of pigs and biodata collection.

A systematic sampling strategy was used to select pigs for inclusion in this study. Because Wambizzi is not a mechanized slaughterhouse and has no slaughter line,^37^ we physically counted animals as they came through the door of the slaughter building and selected every third animal for inclusion in the study.

Selected pigs were ear tagged with a unique identification number, and this number was subsequently used to identify specimens collected from that animal. Biodata was collected using a standard form. The form captured the date of sampling, ear tag number of the pig, rectal temperature (taken when the pig was ear tagged), pig breed (based on visual classification as local, cross, or exotic), sex, whether the male pigs were intact or castrated, visible clinical signs of disease, and source location of the pig (reported to the district level). The biodata form is included in the Supplemental Materials. Sampling occurred over consecutive days, until the sample size for that sampling period was reached.

### Specimen collection and handling.

A panel of samples was collected from each tagged pig. Because *Brucella* spp./*C. burnetii* and *Leptospira* spp. colonize the reproductive tract^33,38^ and kidney,^32^ respectively, these tissues were targeted for molecular detection using qPCR. In pigs, *B. suis* can persist in the uterus^38^ and placenta^33^ in females and the epididymides in males.^33,38^ In livestock, *C. burnetii* colonizes reproductive tissues,^28^ especially the uterus, but little is known about infection in the male reproductive tract.^39^ *Coxiella burnetii* has been isolated from the epididymis tissue in experimentally infected mice.^40^ Although colonization of renal tubules is a feature of chronic leptospiral infection, localization of leptospires in the uterus of pregnant and nonpregnant females and the reproductive tract in males is also common.^18^ Because of the strong association between these pathogens and the reproductive tract, uterus, placenta, and epididymides were collected for qPCR detection of *Leptospira* spp., *Brucella* spp., and *C. burnetii*.

Accordingly, during the evisceration process, a 1 × 1-cm section from the ventral aspect of one kidney and reproductive tissue (uterus/placenta/epididymis) was collected. All samples were placed on ice in an ice box, stored for 2–3 hours until sampling was completed for the day, and then transported to the laboratory at the Makerere University COVAB, Kampala, Uganda, where they were placed under refrigeration until processing the following day. Tissue samples were cut in half, placed in separate cryovials, and stored at −80°C.

### Laboratory analysis.

Reproductive (uterus/placenta/epididymis) and kidney tissues were thawed, and DNA extracted using the DNeasy Blood and Tissue Kit (Qiagen, Germantown, MD). The kit protocol was followed according to manufacturer’s directions, except for the following modifications. After the second wash buffer was added, the collection tubes were centrifuged for 4 minutes. Only 100 µL of elution buffer was added, and incubation time was increased from 1 minute to 3 minutes to increase DNA yield.

#### Detection of pathogenic *Leptospira* spp.

Kidney and reproductive tissues (uterus/placenta/epididymis) were tested for the presence of pathogenic *Leptospira* spp. DNA using the protocol outlined by Smythe et al.,^41^ which targets the rrs (16S) gene and differentiates between pathogenic and nonpathogenic leptospires (detection limit of two cells). This same protocol has been used to detect *Leptospira* infection in bovine kidneys^42^ and equine ocular tissue.^43^ See Table 1 for the primers and probes used in the qPCR protocol. All primers and probes were synthesized by Bio-Rad (Hercules, CA) with a final primer concentration of 0.9 pmol/µL and 0.3 pmol/µL for the probe in the qPCR reaction. For all reactions, 7 µL of template DNA was added to 13 µL of the PCR Master Mix comprising 10 µL IQ Multiplex Powermix (Bio-Rad), 1 µL reagent water, and 1 µL of each of the primer/probe sets (see Table 1), for a total of 20 µL per well. The amplification and florescence detection were conducted in a CFX96 Touch Real-Time PCR Detection System (Bio-Rad) with a program of 3 minutes at 95°C, followed by 45 cycles of 15 seconds at 95°C, and 60 seconds at 60°C. A positive result was assigned if the cycle threshold (Ct) value was < 40 cycles.^41–43^ Positive and negative controls were included on each plate. Positive control DNA for *Leptospira* spp. (specifically *Leptospira canicola*) was sourced from COVAB.

**Table 1:** Primers and probes used for the detection of bacterial zoonoses using quantitative real-time PCR

Pathogen	Forward primer	Reverse primer	Probe	5′ Fluorophore/3′ quencher	Reference
Pathogenic *Leptospira* spp.	CCCGCGTCCGATTAG 3	TCCATTGTGGCCGR^A/G^ACAC	CTCACCAAGGCGACGATCGGTAGC	FAM	41
*Coxiella burnetii*	AAAACGGATAAAAAGCTGTGGTT	CCACACAAGCGCGAT TCAT	AAAGCACTCATTGAGCGCCGCG	CY5	44
*Brucella* spp.	GCTCGGTTGCCAATATCAATGC	GGGTAAAGCGTCGCCAGAAG	AAATCTTCCACCTTGCCCTTGCCATCA	FAM	45
*Brucella abortus*	GCGGCTTTTCTATCACGGTATTC	CATGCGCTATGATCTGGTTACG	CGCTCATGCTCGCCAGACTTCAATG	HEX	
*Brucella melitensis*	AACAAGCGGCACCCCTAAAA	CATGCGCTATGATCTGGTTACG	CAGGAGTGTTTCGGCTCAGAATAATCCACA	TEX	

#### Detection of *C. burnetii*.

Kidney and reproductive tissues (uterus/placenta/epididymis) were tested for the presence of *C. burnetii* DNA using the protocol outlined by Schneeberger et al.^44^ (Table 1). This protocol targets the IS1111 gene of *C. burnetii*. Quantitative real-time PCR was performed in duplex with the *Leptospira* protocol per the reaction conditions described earlier. Positive control DNA for *C. burnetii* was sourced from Vircell, Granada, Spain.

#### Detection of *Brucella* spp.

The reproductive tissue (uterus/placenta/epididymis) was tested for the presence of *Brucella* spp. using the multiplex assay developed by Probert et al.^45^ This protocol contains both genus-level primers/probes as well as primers/probes specific for *B. abortus* and *B. melitensis* (see Table 1). According to this protocol, specimens that are positive using the genus-level primer/probe set and which are deemed negative for both *B. abortus* and *B. melitensis* can be assumed to be *B. suis*. Primers/probe sets were synthesized by Bio-Rad and target the IS711 gene. For all reactions, each well contained 7 µL of template DNA and 13 µL PCR Master Mix comprising 10 µL IQ Multiplex Powermix (Bio-Rad), 1 µL *Brucella* spp. primers/probe set, 1 µL *B. abortus* primers/probe set, and 1 µL *B. melitensis* primers/probe set for a total of 20 µL per well and a final primer concentration of 0.9 pmol/µL and 0.3 pmol/µL for the probe in the qPCR reaction. The amplification and florescence were conducted on the same machine as the *Leptospira* spp./*C. burnetii* assays with a program of 3 minutes at 95°C, followed by 45 cycles of 15 seconds at 95°C and 60 seconds at 57°C. If no amplification occurred by 40 cycles, a negative result was recorded. Positive and negative controls were included on each plate. Positive controls used for the triplex assay included DNA from *B. abortus* (Vircell, Spain), *B. melitensis* 16M Biovar 1 (Friedrich-Loeffler-Institut, Greifswald, Germany), and *B. suis* 1330 Biovar 1 (Friedrich-Loeffler-Institut).

#### *Leptospira* genotyping.

DNA extracted from *Leptospira* qPCR–positive pigs was transported on dry ice to the Sokoine University of Agriculture, Morogoro, Tanzania, for fingerprinting. PCR was conducted with IS1533 primers (EPR-2: CTCGCATCTAACCCACGTTT and EPL-2: AGATTTACTGCTCCGGATGG) and IS1500 primers (iP1: GTTAGCCATGCTTTGAATCGAA and iM16: CGCAGTCGCTGAGTCCTTCTTT) according to protocols detailed by Zuerner et al.^46,47^ These primers have previously been used to identify common *Leptospira* serovars from the East Africa region, confirmed by cross-agglutination absorption tests (CAATs), the gold standard test for identification of serovars.^20,48^ Availability of previous *Leptospira* fingerprints from the region prompted consideration of these primers in the preliminary identification of potential *Leptospira* serovars from the present sample set of qPCR *Leptospira* spp.–positive pigs.

Primers were used individually and in pairs according to Zuerner et al.^46,47^ In brief, the amplification cycles consisted of 35 cycles of 1 minute at 94°C, 2 minutes at 51°C, and 2 minutes at 72°C. PCR products were visualized in 1.5% gel electrophoresis.

### Data analysis.

Pig characteristics collected at the time of sampling included the date of sampling, breed, sex, rectal temperature, source location, and clinical signs. These data were checked for typographical errors before being imported into SPSS 24.0 (IBM Corp., Armonk, NY) for analysis. Two new variables were created, namely, season and region, based on the date of sampling and source location, respectively. Summary statistics calculated included the frequency of the characteristics of pigs sampled and the distribution of qPCR-positive cases between tissue types analyzed and pig characteristics. Univariable logistic regression was performed to determine if any of these explanatory variables were associated with qPCR status. Explanatory variables with *P*-value of < 0.15 were included in a multivariable logistic regression model. The model was built using backward stepwise regression, and each model was tested for goodness of fit to the data using the Hosmer and Lemeshow test (H&L). Three-way and two-way interactions between explanatory variables were tested using the interaction term as part of the model building. Interactions that were statistically significant (*P* < 0.05) were retained in the model. Collinearity among explanatory variables was assessed using a chi-square test (χ^2^). A pair of variables was considered highly correlated if the chi-square test statistic was ≤ 0.05.

Source locations (reported to the district level) were entered into Microsoft Excel and checked for spelling accuracy. The districts were joined to the centroid of each district polygon in the 2014 Global Administrative Unit Layers for Uganda (Food and Agriculture Organization, Rome, Italy) using ArcGIS 10.2 (Environmental Systems Research Institute, Redlands, CA). The number of qPCR positive pigs in each district was mapped and, along with sampling period, symbolized using unique values.

To investigate correlation of rainfall and leptospiral prevalence, historical (1970–2000) monthly rainfall data were downloaded from WorldClim (www.worldclim.org). In ArcGIS, the rainfall raster files were converted to points and then joined to the Uganda district polygons and average rainfall by district calculated. Correlation between average district rainfall and leptospiral prevalence by sampling month was calculated using Spearman rank correlation in SPSS.

## RESULTS

### Study population.

Table 2 shows the characteristics of the 649 pigs that were sampled at Wambizzi between December 2015 and October 2016. Fifty-seven percent (*n* = 372) of the pigs sampled were female. Thirty-eight percent (*n* = 247) of the pigs were crossbreed, 34.2% (*n* = 222) were exotic breed, and 25.7% (*n* = 167) were local breed. At the time of sampling, rectal temperatures ranged from 34.2°C to 42.1°C (mean: 38.6°C, SD: ±0.91°C). Forty-five (6.9%) of the pigs had a temperature above 39.8°C when they were sampled. Dullness (*n* = 7, 1.1%), diarrhea (*n* = 6, 0.9%), and skin flash or rash (*n* = 5, 0.7%) were the most frequently observed clinical signs. Pig traders reported that 53.9% (*n* = 350) of the pigs were sourced from the Central region and 17.1% (*n* = 111) from the Eastern region.

**Table 2:** Frequency of pathogenic *Leptospira* spp. by pig characteristics based on data collected at the Wambizzi Cooperative Society, Kampala, Uganda, 2015–2016 (*n* = 649)

	Total		Pathogenic *Leptospira* spp. positive		Pathogenic *Leptospira* spp. negative	
	*N*	Percentage (%)	*N*	Percentage (%)	*N*	Percentage (%)
Total	649	100	68	10.5	581	89.5
Sex						
Female	372	57.3	49	7.6	323	49.8
Male	267	41.1	18	2.8	249	38.7
Missing	10	1.6	1	0.2	9	1.4
Male status						
Intact	92	34.6	10	3.7	82	30.7
Castrated	174	65.4	8	3.0	166	62.3
Breed						
Cross	247	38.1	23	3.5	224	34.5
Exotic	222	34.2	23	3.5	199	30.7
Local	167	25.7	21	3.2	146	22.5
Missing	13	2.0	1	0.2	12	1.8
Fever at time of sampling						
Febrile (> 39.8°C)	45	6.9	5	0.8	40	6.2
Afebrile (< 39.8°C)	594	91.5	60	9.2	534	82.3
Missing	10	1.6	3	0.5	7	1.1
Clinical signs observed (visual inspection)						
Dullness	7	1.1	1	0.2	6	0.9
Diarrhea	6	0.9	0	0	6	0.9
Skin flash	5	0.7	0	0	5	0.8
Shivering	3	0.5	0	0	3	0.5
Anorexia	2	0.3	0	0	2	0.3
Vomiting	1	0.2	0	0	1	0.2
Hind limb paralysis	1	0.2	0	0	1	0.2
Lice infestation	1	0.2	0	0	1	0.2
Tick infestation	1	0.2	0	0	1	0.2
Region pigs were sourced from						
Central	350	53.9	46	7.1	304	46.8
Eastern	111	17.1	6	0.9	105	16.2
Northern	14	2.2	1	0.2	13	2.0
Western	24	3.7	2	0.3	22	3.4
Missing	150	23.1	13	2.0	137	21.1
Season of sampling						
Dry	322	49.6	31	4.8	291	44.8
Rainy	327	50.4	37	5.7	290	44.7
Sampling period						
December 2015	162	24.9	18	2.8	144	22.2
March 2016	160	24.7	32	4.9	128	19.7
June 2016	160	24.7	13	2.0	147	22.7
October 2016	167	25.7	5	0.8	162	25.0

### Prevalence of bacterial pathogens.

**Figure 1.**
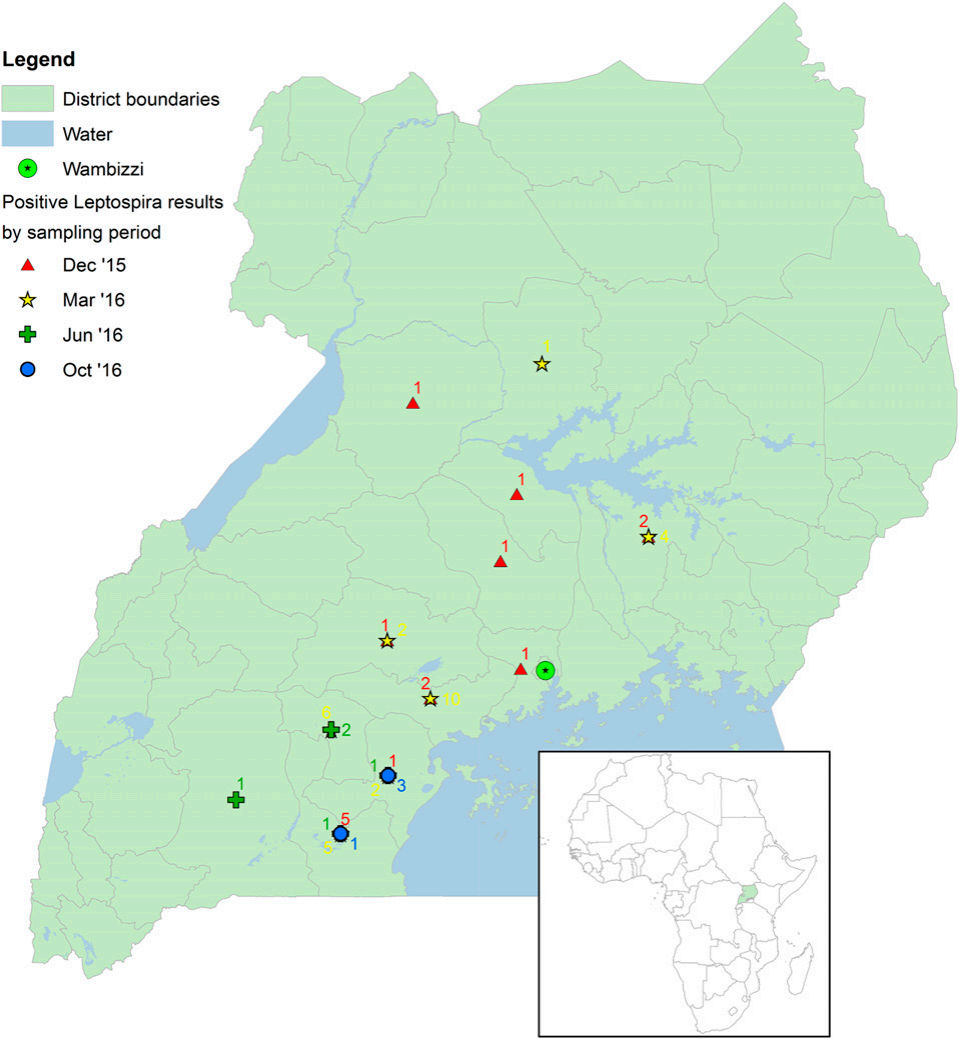
Source locations of *Leptospira* quantitative real-time PCR–positive pigs sampled at the Wambizzi Cooperative Society slaughterhouse, Kampala, Uganda, 2015–2016.

Table 2 reports the frequency of pathogenic *Leptospira* spp. by pig characteristics. A total of 649 pigs had kidney (*n* = 645) and reproductive tissues (*n* = 443) analyzed using qPCR. Sixty-eight pigs (10.5%) were positive for pathogenic *Leptospira* spp. (see Figure 1). The Ct values ranged from 32.03 to 39.63, with an average Ct value of 35.26. Thirty-two kidneys (5.0%) and 39 (8.8%) reproductive tissues (uterus, placenta, or epididymis) were positive. Both kidney and reproductive tissues from three pigs (4.4%) were positive. None of the pigs were positive for *Brucella* spp. or *C. burnetii* using qPCR.

### Risk factors for *Leptospira* infection.

In univariable analysis, pig sex, sampling period, and region the pig was sourced from were all significantly associated with qPCR-positive *Leptospira* cases. Region and sampling period were highly correlated (*P* = 0.03). Region was removed from the final multivariable model because seasonality is a known risk factor for leptospiral infection, and during model building, pig sex and sampling period explained more of the variance in the outcome, qPCR *Leptospira* status, than the model that included region, sampling period, and pig sex (log likelihood ratio: 317.44, χ^2^ = 28.14, df = 4, *P* < 0.01, H&L *P* = 0.50 versus log likelihood ratio: 312.94, χ^2^ = 32.64, df = 7, *P* < 0.01, H&L *P* = 0.14, respectively). Therefore, in the final multivariable model (Table 3), pigs sampled in March 2016 (odds ratio [OR]: 2.23; 95% CI: 1.15,4.33; *P* = 0.02) and October 2016 (OR: 0.30; 95% CI: 0.10,0.93; *P* = 0.04) were more and less likely to be *Leptospira* positive than pigs sampled in December 2016, respectively. In addition, female pigs were more than twice as likely to be *Leptospira* qPCR positive (OR: 2.37; 95% CI: 1.25,4.48; *P* < 0.01) than male pigs. There was no significant correlation between average district rainfall and leptospiral prevalence by month of sampling, although in the month of December the correlation was moderate and positive (rho = 0.43, *P* = 0.11).

**Table 3:** Risk factors for pathogenic *Leptospira* spp.–positive status in 649 pigs sampled at the Wambizzi Cooperative Society, Kampala, Uganda, 2015–2016

Explanatory variable	Outcome variable: pathogenic *Leptospira* spp. positive in the kidney or reproductive tissue				
	Frequency (%)	Unadjusted OR (95% CI)	*P*-value	Adjusted OR (95% CI)	*P*-value
Sex					
Female	49 (7.6)	2.10 (1.19,3.69)	0.01	2.37 (1.25,4.48)	< 0.01
Male	18 (2.8)	1.00	–	1.00	–
Breed					
Cross	23 (3.6)	0.71 (0.38,1.34)	0.29	–	–
Exotic	23 (3.6)	0.80 (0.43,1.51)	0.50	–	–
Local	21 (3.3)	1.00	–	–	–
Fever at the time of sampling					
Febrile (> 39.8°C)	5 (0.8)	1.11 (0.42,2.93)	0.83	–	–
Afebrile (< 39.8°C)	60 (9.4)	1.00	–	–	–
Region					
Central	46 (9.2)	1.00	–	*	–
Eastern	6 (1.2)	0.38 (0.16,0.91)	0.03	–	–
Northern	1 (0.2)	0.51 (0.07,3.98)	0.52	–	–
Western	2 (0.4)	0.60 (0.14,2.64)	0.50	–	–
Season					
Rainy season	37 (5.7)	1.20 (0.72,1.98)	0.48	–	–
Dry season	31 (4.8)	1.00	–	–	–
Sampling period					
December 2015	18 (2.8)	1.00	–	1.00	–
March 2016	32 (4.9)	2.00 (1.07,3.74)	0.03	2.23 (1.15,4.33)	0.02
June 2016	13 (2.0)	0.71 (0.33,1.50)	0.37	0.59 (0.21,1.70)	0.33
October 2016	5 (0.8)	0.25 (0.09,0.68)	0.01	0.30 (0.10,0.93)	0.04

OR = odds ratio. Explanatory variables with *P* < 0.15 in the univariable analyses were included in the final multivariable logistic regression model.

* This variable was removed from the final multivariable model. See Results section for description.

### *Leptospira* serovars identified from DNA fingerprinting.

**Figure 2.**
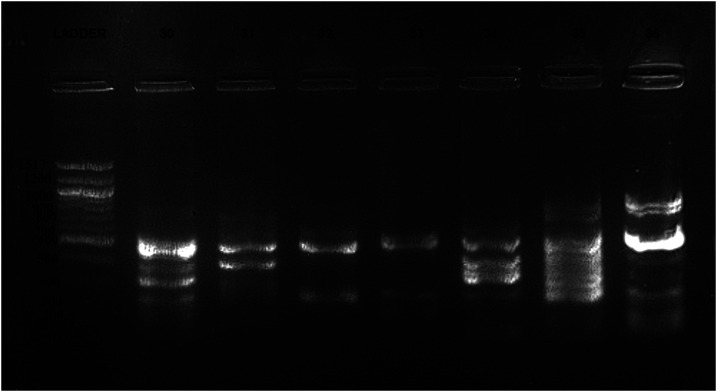
Fingerprinting with EPR-2 primer suggesting four *Leptospira* serovar clusters: serovar 1 (samples 30 and 34), serovar 2 (sample 31), serovar 3 (samples 32 and 33), and serovar 4 (35 and 36).

Positive *Leptospira* fingerprinting was obtained from 43 (60.5%) of the 71 qPCR-positive pig samples. Findings suggest existence of at least four *Leptospira* serovars or strains based on IS1533 primers (see Figure 2). Samples identified as belonging to *Leptospira* serovar Kenya and other unknown serovars are shown in Table 4*.* Fingerprinting with IS1533 primers produced more positive bands than the IS1500 primers (not shown).

**Table 4:** Affiliation of samples to candidate *Leptospira* serovars based on DNA fingerprinting

Sample ID	Potential serovar	Locality
23	Kenya	Rakai district
26	Kenya	Mpigi district
31	Kenya	Unknown
32	Kenya	Rakai district
33	Kenya	Sembabule district
59	Kenya	Sembabule district
1	Serovar 2	Wakiso district
6	Serovar 2	Kyotera district
24	Serovar 2	Mubende district
27	Serovar 2	Buyende district
29	Serovar 2	Lwengo district
3	Serovar 3	Mubende district
28	Serovar 3	Lwengo district
58	Serovar 3	Unknown
69	Serovar 3	Lyantonde district
25	Serovar 4	Mpigi district
30	Serovar 4	Gomba district
34	Serovar 4	Unknown
35	Serovar 4	Isingiro district
36	Serovar 4	Unknown

## DISCUSSION

This is the first large-scale epidemiological study using molecular detection methods to investigate leptospirosis, brucellosis, and Q fever (coxiellosis) in pigs in East Africa. Ten percent of pigs had pathogenic leptospiral DNA in the kidney or reproductive tissue. *Brucella* spp. and *C. burnetii* were not detected. Detection of pathogenic leptospires in kidney and reproductive tissues suggests the bacteria are likely to be excreted via urine and reproductive fluids into the environment. This poses a potential occupational hazard to slaughterhouse workers and pig farmers who encounter stillborn and aborted fetuses. Environmental contamination from the shedding of leptospires in urine is an important source of infection for humans and other animals.^18^

Pigs sampled in March 2016 were more likely to be *Leptospira* positive than those sampled in December 2015. This is consistent with ecological factors associated with the increased incidence of leptospirosis. Warm, wet weather favors the persistence and replication of leptospires.^49^ Uganda experiences bimodal rainfall. March to June is typically the season of heavy rains, particularly in Central region, where the frequency of *Leptospira*-positive pigs was higher than other regions. The Central region has the highest pig population and largest average herd size in the country.^14^ In addition, more intensive pig keeping systems occur in peri-urban and urban areas,^50,51^ which are found throughout the Central region because of its proximity to the capital city, Kampala. The warm, wet weather and higher pig densities found in the Central region are both factors that favor the epidemiology of leptospirosis in pigs. Although there was no positive correlation between district level rainfall and leptospiral prevalence in March, the analysis was likely limited by the number of locations (*n* = 9 districts) with positive pigs in March.

It is not fully understood why pigs sampled in October 2016 were less likely to be *Leptospira* positive than those sampled in December 2015. October through December is the second rainy season, though these rains are intermittent as compared with the heavy rains of March to June. High season rainfall is often associated with leptospirosis,^49^ and the intermittent rains in October 2016 may not be ideal ecological conditions for the persistence and transmission of leptospires from the environment. There may also be differences in the distribution of reservoir hosts during this time which impacts the transmission of infective leptospires to pigs.

In this study, we also found that female pigs were 2.37 times more likely to be *Leptospira* qPCR positive than males. In female pigs, leptospiral infection causes stillbirths, abortions, and infertility.^52–55^ Given this impact on reproductive performance in sows, it can be postulated that female pigs experiencing clinical signs consistent with leptospiral infection may be culled (sold to slaughterhouses), as they are no longer economically viable in smallholder farming systems. This could explain why females in this study were significantly more likely to be *Leptospira* positive. Furthermore, most of the male pigs in this study were castrated which precluded testing of the reproductive tissues of these animals. Frequency of leptospires in reproductive tissues was slightly higher than that in kidneys (8.8% and 5%, respectively). Testing of intact males on farms may reveal higher prevalence. As we were unable to determine the age of pigs sampled, it is also possible that the association between pig sex and *Leptospira*-positive status to be confounded by age. However, previous studies have shown that age is not associated with seropositive status^56^ or clinical signs.^57^

We identified four *Leptospira* serovars or strains circulating in pigs in this study. Several serovars are associated with infection in pigs. Studies in Africa have shown the most common serovars circulating in pigs are celldoni in Zambia,^58^ butembo in Ethiopia,^59^ ballum in Tanzania,^60^ icterohaemorrhagiae and hardjo in South Africa,^61^ and pomona in Uganda^62^ and Egypt.^63,64^ Some samples in the present study had patterns similar to serovar Kenya reported in the African giant pouched rats^65^ and other rodent species^48^ and pigs^20^ in neighboring Tanzania. Genotyping of serovar Kenya isolates revealed it belongs to several species: *Leptospira interrogans*, *Leptospira borgpetersenii*, and *Leptospira kirschneri*.^66^ Another three distinct fingerprinting patterns were obtained from pigs for which local isolates were not available for comparison. The absence of suitable sequencing facilities and budgetary constraints made the use of more robust molecular methodologies for serovar identification impractical. Although this study failed to determine more specific *Leptospira* serovars, fingerprinting suggests preliminary existence of more than one serovar; information which may prove useful for control interventions were the ecologies of these serovars to be established. These findings call for further studies aiming for isolation of live leptospires to enable proper taxonomical identification by the CAAT. Although vaccination of pigs is not routinely practiced in Uganda, if circulating serovars were known, vaccination could be implemented as part of leptospirosis control and prevention measures.

The choice to sample reproductive tissues in this study may mean we missed *Brucella* spp. and *C. burnetii*. In rare cases, *Brucella* spp. and *C. burnetii* can be found in the spleen, lung, liver, and lymphatic tissue,^28,33^ but it is unusual in those cases not to also find the pathogens in the reproductive tissue. Furthermore, failure to detect *Brucella* spp. or *C. burnetii* is consistent with the limited published studies in the region which suggest low prevalence of these organisms in pigs. A few studies undertaken decades ago confirm exposure of pigs in Africa to *C. burnetii*,^67,68^ but principal livestock reservoir species are thought to be sheep, goats, and cattle.^2,39,69^ Similarly, serological evidence for brucellosis in pigs in Africa exists.^25,36,70–74^ In Uganda, 10% of the porcine samples submitted to referral laboratories were *Brucella* seropositive,^71^ although it is unclear what serological method was used to screen the pig samples. Two other serological studies found a 0%^75^ and 0.1%^36^ seroprevalence in pigs in Uganda. Based on this research, the risk of acquiring brucellosis or Q fever from pigs or their products in Uganda is likely negligible.

This initial study detecting pathogenic leptospires in pigs raises several research priorities for future studies. The economic impact of leptospirosis on pig production needs to be assessed to determine its priority in comparison to more recognized pig diseases in Uganda. Elsewhere, chronic leptospirosis infections in pigs have been found to lead to an array of reproductive disorders, including abortions, neonatal mortality, premature births, and stillbirths, with differences noted between serovars.^55^ Because leptospirosis is a zoonotic disease, its impact on human health must also be identified and understood. Public awareness of leptospirosis, particularly to high-risk populations such as farmers and slaughterhouse workers, is needed. In addition, to understand how to control and prevent infection, reservoirs of leptospires must be identified as well as the circulating serovars. This can only be achieved with isolation of leptospires from a wide range of suitable hosts found in Uganda. Finally, to safeguard livestock farmer’s livelihoods, the health of the citizens of Uganda, and food and water security, leptospirosis demands more prominent awareness in the medical and veterinary communities.

## CONCLUSION

This is the first study to report infection of pigs by pathogenic *Leptospira* spp. in Uganda. Using qPCR, more than 10% of pigs had leptospiral DNA in the kidney or reproductive tissue. This indicates that infection of pigs with pathogenic *Leptospira* spp. is relatively common and suggests the disease may have a currently unrecognized impact on the pig sector, both in terms of pig productivity and occupational risks to pig farmers, pig traders, and slaughterhouse workers. DNA fingerprinting identified at least four distinct serovars infecting pigs.

## Supplemental materials

Supplemental materials
