# Supplementary material for: Selected Endemic Zoonoses in Pigs Presenting for Slaughter in Kampala, Uganda
Source: Am J Trop Med Hyg. 2020 Oct 12;103(6):2552–60. doi: 10.4269/ajtmh.20-0033 (PMC7695076; doi:10.4269/ajtmh.20-0033)
Supplement: Supplementary file 1 [file tpmd200033.SD1.pdf]

INTERNATIONAL LIVESTOCK RESEARCH INSTITUTE

THE UNIVERSITY OF SYDNEY

Wambizzi Sampling: Pig Biodata

1. Date (DD/MM/YY): ___/___/___

2. Ear tag ID (identical to sample ID whole blood, serum, fecal sample, nasal swab, urine sample): ____________

3. Rectal temperature °C __._

4. Pig breed: local ☐

   exotic ☐

   cross ☐

5. Gender: male ☐ male intact Y / N

   female ☐

6. Visible symptoms of disease (circle all that apply):

1=diarrhoea	2= anorexia	3=dullness	4=swaying gait	5=skin flash	6=respiratory problems
7=vomiting	8=coughing	9= shivering	10=foaming at mouth	11=wounds	12=Other

If other, please explain:

___

___

7. Pig source location (village, subcounty, district):

___

8. Gross pathology noted:

___

___

___

___

Barcode for:

a. epididymis sample___________________________
b. liver sample________________________________
c. lung sample_________________________________
d. placenta sample_____________________________
e. spleen sample_______________________________
f. Tracheobronchial lymph nodes________________
